# Parenting style and family type, but not child temperament, are associated with television viewing time in children at two years of age

**DOI:** 10.1371/journal.pone.0188558

**Published:** 2017-12-20

**Authors:** Anna S. Howe, Anne-Louise M. Heath, Julie Lawrence, Barbara C. Galland, Andrew R. Gray, Barry J. Taylor, Rachel Sayers, Rachael W. Taylor

**Affiliations:** 1 Department of Medicine, University of Otago, Dunedin, New Zealand; 2 Department of Human Nutrition, University of Otago, Dunedin, New Zealand; 3 Department of Women’s and Children’s Health, University of Otago, Dunedin, New Zealand; 4 Department of Preventive and Social Medicine, University of Otago, Dunedin, New Zealand; Swansea University, UNITED KINGDOM

## Abstract

**Objectives:**

Despite the American Academy of Pediatrics (AAP) recommending that electronic media be avoided in children under two years of age, screen use is common in infants and toddlers. The aims of this study were to determine how parenting style, infant temperament, and family type are associated with television viewing in two-year-old children.

**Study design:**

Participants were from the Prevention of Overweight in Infancy (POI) randomized controlled trial (n = 802) (Dunedin, New Zealand). Demographic information was collected at baseline (late pregnancy), and television and other screen time assessed by questionnaire at 24 months of age. Parenting style (Parenting Practices Questionnaire), infant temperament (Colorado Childhood Temperament Inventory), and family type (7 categories) were reported by both parents.

**Results:**

Data were available for 487 participants (61% of the original participants). Median television viewing was relatively low at 21 minutes per day, or 30 minutes in those watching television (82%). Children who watched television played with mobile phones (12% of children) or iPads/tablets (22% of children) more frequently than children who did not (6% of children). In terms of parenting style, children of more authoritarian mothers (β = 17, 95% CI: 6–27 minutes), more authoritarian partners (β = 14, 95% CI: 2–26 minutes), or more permissive mothers (β = 10, 95% CI: 3–17 minutes) watched significantly more television. No significant relationships were observed between child temperament and time watching television after adjustment for confounding variables. Children from “active” families (as rated by partners) watched 29 minutes less television each day (*P* = 0.002).

**Conclusions:**

Parenting style and family type were associated with television viewing time in young children, whereas child temperament was not.

## Introduction

Energy-dense foods, low levels of physical activity, and sedentary behaviors (including use of electronic media such as television) have all been implicated in childhood obesity [1, 2]. Independent of obesity, childhood “screen time” is also associated with an increased risk of sleep disturbances [3] and problems with attention [4], and may displace time in other developmentally important activities including reading [5] and creative play [6]. Despite clear recommendations that parents should discourage media use, including television viewing, in children less than two years of age [7, 8], it is clear that many infants and toddlers [9], perhaps as many as 90% [10], are exposed to television and other electronic media. Limited evidence suggests New Zealand toddlers may be watching as little as 1–3 hours per week [11] or as much as 90 minutes per day [12].

Given this high exposure, it is surprising that relatively few studies have examined factors that may predict levels of television viewing in young children [13]. Higher viewing hours have been associated with ethnic minority status, lower levels of maternal education and employment, marital status, and maternal depression in some [10, 14, 15], but not all [16, 17], studies in infants and preschool children. Parenting behaviors, such as allowing a television in the child’s bedroom [18, 19], fewer rules around television use [20], and greater maternal television viewing [17, 21, 22], have also been associated with higher viewing time in children, although these findings have not been unanimous [13].

Investigation of wider issues, including parenting style and family “type”, is warranted given the over-riding influence of the home environment in the early years [23]. In one study, low-income families in which parents valued complete compliance had pre-school children who watched more television [15], but other studies have observed no relationship between parenting style and screen use [19, 22]. Infant temperament may be important if parents of young children with more challenging behaviors use screen time as a distraction [24], for instance, it has been reported that infants whose mothers perceived them to be fussy or cry frequently, were more likely to watch television daily [17]. Both parenting style [25] and ratings of infant temperament [26] can differ between mothers and fathers, yet no studies appear to have evaluated media viewing in young children in relation to these characteristics assessed separately in both parents.

Whilst television viewing has previously been the main source of screen use, computers, gaming consoles, smart phones, and tablets are increasingly popular [27]. In particular, the rise of touchscreen devices which are small and relatively easy to use can facilitate engagement by the very young [27]. One survey of infants and toddlers suggests that as many as 50 percent of children under 2 years of age could be allowed to use touch screens by their parents [28]. Some parents believe that touchscreen devices teach the child new skills and knowledge, are fun and entertaining, or occupy the child when the parent needs to do something [28]. However, in children 3–5 years of age, while touchscreen devices were found to be used for educational games and apps, traditional television viewing was still more common, making up 60% of the reported viewing time [29]. Concurrent screen use is common in adolescents and young adults, and 40% of pre-schoolers have been found to multitask with screen devices [29]. While the amount of time the children spent using touchscreen devices was positively associated with concurrent screen use, preference for television viewing was unrelated to concurrent device use [29].

Therefore, the aim of this study was to examine the extent to which parenting style, infant temperament, and family type, as estimated separately by mothers and their partners, were associated with screen time in two-year-old children. In addition, it was of interest to us to see what associations might exist between the amount of time a toddler watches television and the use of other media.

## Methods

These data were obtained from the Prevention of Overweight in Infancy (POI) study, a randomized controlled trial investigating whether extra education and support around food, activity, breastfeeding and sleep reduced rates of excessive weight gain in the first two years of life (clinical trials registration at ClinicalTrials.gov (Clinical Trials NCT00892983)). As a protocol paper has been published [30], only methods relevant to the current study are described briefly here. The New Zealand Lower South Regional Ethics Committee approved the study (LRS/08/12/063), and all adult participants gave written informed consent for themselves and their children. A cohort of 802 women were recruited in late pregnancy for the two-year intervention and randomized to one of four groups: 1) control, 2) FAB (food, activity, breastfeeding), 3) Sleep or 4) Combination (both interventions). Those in the FAB and Combination groups received a total of 8–9 contacts, covering a wide range of topics, including education on restricting television (and other media) viewing in children before two years of age. Those in the Sleep (and Combination) groups received an educational programme (2 sessions) discussing normal sleep patterns, safe sleep practices and prevention of sleep problems. All participants received standard government-funded well-child care (http://www.wellchild.org.nz/). There were no significant differences among the intervention groups in the time spent watching television at 24 months [31]. Therefore, data from all four groups were combined in the current analysis, and intervention group was included as a confounder in analyses as described below.

Data collected at baseline (third trimester) included estimated maternal pre-pregnancy body mass index (BMI), current estimated paternal BMI, and maternal and partner (all fathers for this sub-study population) age and ethnicity. The level of household deprivation was provided by the New Zealand Deprivation Index (NZDep2006), which ranges from 1 (indicating areas of least deprivation) to 10 (areas of highest deprivation) [32]. Infant birth characteristics (date of birth, weight, gestational age) were collected from hospital records. Maternal depression was assessed using the Edinburgh Postnatal Depression Scale (EPDS) [33], validated for use in the prenatal period [34]. Scores were dichotomized into ‘not depressed’ (< 10), and ‘possibly depressed’ (≥ 10).

Data collected when the children were 24 months of age included anthropometry, parent and child media usage, parenting style, child temperament, family type, and maternal and child physical activity. Weight (WB-100MA, Tanita (kg)) and height (Holtain Model 603VR, Harpenden and Leicester Height Measure Mk II, Invicta (cm)) were measured in duplicate with the subject dressed only in light clothing (diaper and singlet for children, minimal clothing for adults) following standard techniques [35] in both parents and in the child. Child body mass index (BMI) z-score was calculated using World Health Organization growth standards [36]. Mothers reported their own usual screen time (television, computers and tablets, video games) in minutes per day. Mothers provided child viewing time (television, DVDs and videos) as both frequency (days per week) and time (usual minutes per day on days watched), from which minutes of viewing per day was calculated (this was the variable used to describe television watching in subsequent hypothesis testing). In addition, mothers provided frequency of child media use (television, DVDs or videos, computer, mobile phone, and tablet use) categorized as: does not use, occasional (up to 2 times per week), regular (3–5 times per week), and daily (every day). Maternal physical activity was assessed using the short form of the New Zealand Physical Activity Questionnaire which is based on the International Physical Activity Questionnaire [37], so that metabolic equivalents (MET) minutes per week could be calculated [38]. Physical activity was assessed in children using Actical accelerometers (Mini-Mitter Co., Bend, OR) worn over the right hip 24-hours a day for 5–7 days, and expressed as counts per minute once all sleep time had been removed individually from the data using an automated algorithm [39, 40]. Parenting style was assessed in mothers and fathers using a 30-item questionnaire which provided scores for three sub-scales: authoritarian (13-items), authoritative (13-items), and permissive (4-items) parenting, assessed on a six-point scale (never to always) [41]. Dimensions of authoritative parent style were assessed using statements about responsiveness to child’s feelings/needs, encouraging child to speak freely, and treating child as an equal member of family. Dimensions of authoritarian parenting were assessed with statements about reminding the child I am the parent, using threats as a form of punishment, and criticism to improve behaviour, while dimensions of permissive parenting was assessed with statements about finding it difficult to discipline the child, and ignoring bad behaviour. Both parents also completed the 30-item Colorado Childhood Temperament Inventory [42] which yields six sub-scales: sociability (prefers the presence of others to being alone), emotionality (tendency to get easily distressed and upset), activity (activity level), attention span-persistence (distractability), reaction to food (extent to which a new food is taken without fussing), and soothability (ease with which the infant can be calmed when upset). Cronbach’s alpha for all subscales in our study population ranged from 0.98–1.00. Each parent was also asked to indicate the extent to which they felt their family was i) “active or sporty”, ii) “media savvy”, iii) “bookish”, iv) “outdoor people”, v) “musical”, vi) “religious/spiritual”, and vii) “creative or arty” using possible response options of “not really us”, “a little like us”, or “definitely like us”.

### Statistical analysis

“Minutes of television per day” was used to examine whether children met the 2011 AAP guideline [7]: no television was coded as meeting the guideline, and any minutes of television was coded as not meeting the guideline. While the 2016 AAP guidelines [8] recommend avoiding digital media use for children younger than 18–24 months, we have categorized television use based on the 2011 AAP guidelines of discouraging media use in those younger than 2 years [43]. To compare differences between groups, Chi-square tests or Fisher’s exact test (where more than 20% of cells had expected frequencies below 5) were used for categorical variables with pair-wise comparison undertaken for categorical variables with more than two categories if there was evidence of a significant difference between the categories overall. Mann-Whitney tests were used for continuous or ordinal variables. As the continuous data were non-normally distributed, they are presented as medians with inter-quartile ranges (IQR). Due to the non-normal distribution of the residuals, quantile regression using the 50^th^ percentile (median) was used to investigate associations between demographic variables and television minutes, and to examine the unadjusted and adjusted associations between independent variables (parenting style, infant temperament, family type) and television minutes. Continuous independent variables were tested for nonlinearity by adding a quadratic term (none were significant). Quantile regression models were adjusted for intervention group, demographic variables from the unadjusted models that had *P* ≤ 0.25, and household deprivation and maternal parity (stratification variables used when randomizing participants to the POI study groups). Although children’s physical activity reached the statistical threshold for inclusion in the models, it was not included in the fully adjusted models because the limited number of children with accelerometry data would have reduced the number of participants available for the analysis. Investigation into the missing data indicated that those with missing data did not differ with those who had complete data in respect to demographic variables. Some missingness was due to erroneous data discovered during data cleaning, and some was because some participants refused to take part in the collection of the physical activity data. These missing data were not imputed because imputing data ‘missing not at random’ can introduce more bias when using multiple imputation than complete case analysis [44]. Wald tests were used to determine the overall effect for categorical variables in the regression models, with pairwise comparisons between different levels of the variable conducted for categorical variables with more than two categories if there was evidence of a significant difference between the categories overall. The effect sizes for quantile regression models are the predicted change (95% CI) to median minutes of children’s television viewing per day for every unit change in the independent variable, or between reference and non-reference levels of categorical variables.

Two-sided *P*-values less than 0.05 were considered statistically significant. All statistical analyses were conducted using Stata 12.1 (StataCorp, College Station, TX, USA).

## Results

**Fig 1:**
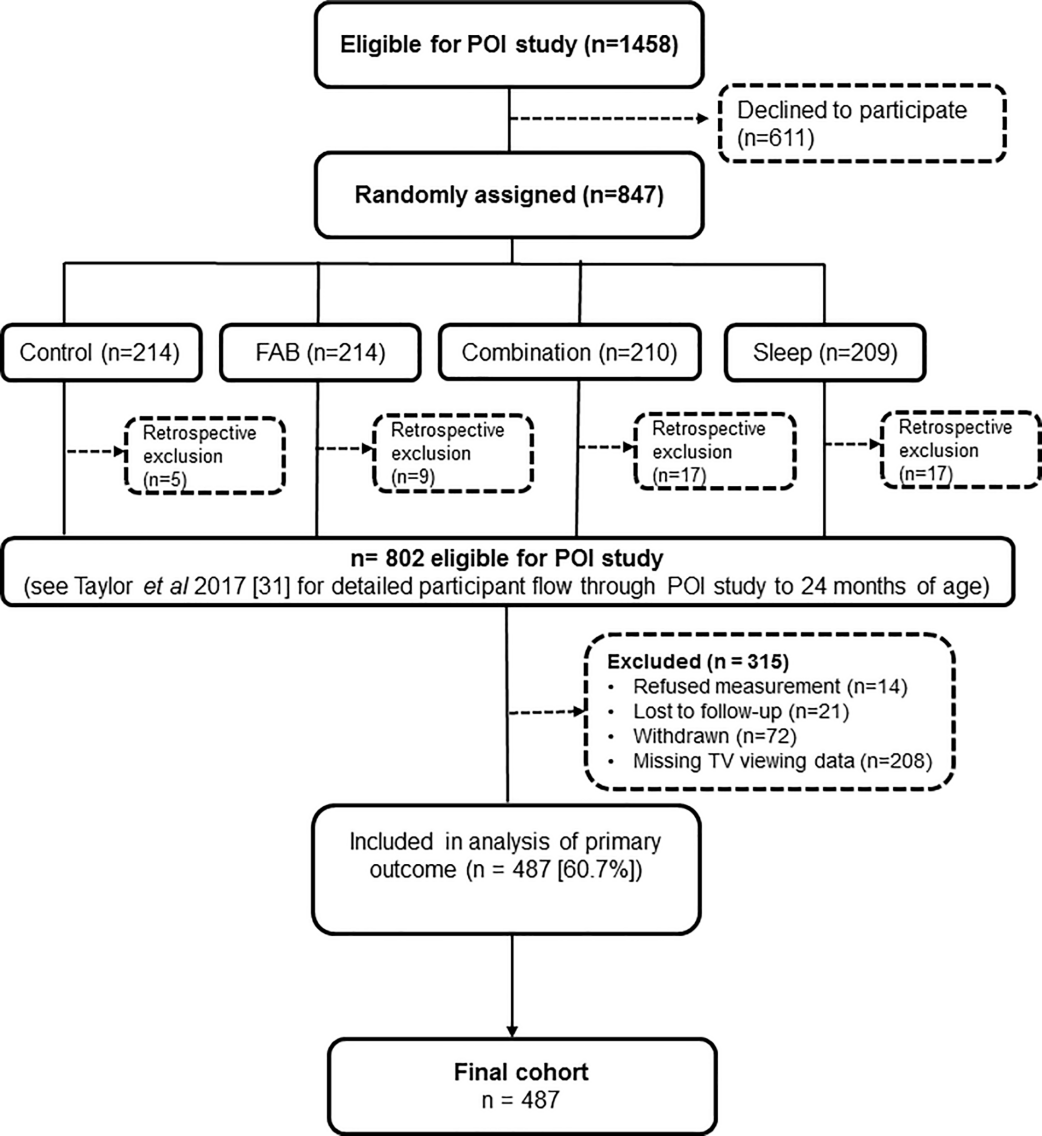
Flow of POI study participants through to 24 months of age.

At 24 months of age, 686 participants remained in the POI study (86%), although only 487 families provided questionnaire data (61% of the original participants) (**Fig 1)**.

**Table 1** demonstrates that the only differences between participants who had usable data and those who did not were: maternal and partner age (those with useable data were older), and partner ethnicity (those with useable data were more likely to be New Zealand European).

**Table 1:** Characteristics of the study sample at baseline (n = 802).

	Did provide data (n = 487)		Did not provide data (n = 315)			
	n (%)		n (%)		P*	
***Data presented as n (%)***						
Child Sex					0.158	
*Female*	230 (47%)		161 (51%)			
*Male*	257 (53%)		154 (49%)			
Child ethnicity					0.623	
*New Zealand European*	385 (79%)		240 (76%)			
*Māori*	43 (9%)		32 (10%)			
*Other*	59 (12%)		43 (14%)			
Maternal ethnicity					0.098	
*New Zealand European*	424 (87%)		258 (82%)			
*Māori*	22 (5%)		24 (8%)			
*Other*	41 (8%)		33 (10%)			
Partner ethnicity					**<0.001**	
*New Zealand European*	337 (69%)		149 (47%)		<0.001	
*Māori*	24 (5%)		12 (4%)		0.490	
*Other*	126 (26%)		154 (49%)		<0.001	
Household deprivation^†^					0.065	
*1–3 (Low)*	180 (37%)		96 (31%)			
*4–7*	209 (44%)		141 (45%)			
*8–10 (High)*	91 (19%)		77 (24%)			
***Data presented as median (IQR)***^‡^	**n**	**Median (IQR)**	**n**	**Median (IQR)**		
Child minutes of TV watching at 24 months	487	21.4 (54.3)	-	-	-	
Maternal age at birth (y)	487	32.5 (6.3)	314	30.4 (9.2)	**<0.001**	
Partner age at birth (y)	401	34.4 (7.7)	180	33.6 (9.2)	**0.026**	
Child BMI z-score at 24 months	478	0.8 (1.2)	205	0.7 (1.2)	0.087	
Maternal pre-pregnancy BMI (kg/m^2^)**	487	23.7 (6.1)	312	24.5 (5.9)	0.380	
Maternal BMI at 24 months (kg/m^2^)**	371	25.5 (7.0)	151	26.6 (7.4)	0.101	
Partner BMI at 24 months (kg/m^2^)**	363	26.9 (5.6)	120	26.8 (6.0)	0.449	
Child physical activity at 24 months (cpm)	264	270 (107)	38	285 (119)	0.545	

* Chi-squared, or Fisher’s Exact when appropriate, test for categorical variables, Wilcoxon-Mann-Whitney test for continuous variables. Pair-wise comparisons were undertaken for categorical variables with more than two categories only where there was evidence of a significant difference between the categories overall.

^†^ Salmond C. *et al* [32].

^‡^ Data not normally distributed, thus presented as median (IQR).

** Maternal pre-pregnancy BMI was self-reported BMI, maternal BMI at 24 months was measured, Partner BMI at 24 months was measured.

Minutes of television watched did not differ between the four study groups and ranged from 0 (18% of children) to 120 minutes per day, with median (IQR) viewing of 21 (IQR 54) minutes per day in the total sample (**Table 1**), and 30 (IQR 46) minutes in those who watched television. In our sample, most children watched DVDs or videos (75%), television (76%) and played games on computers and gaming consoles (73%) at least once a week, and a number of children were using mobile phones (17%), and tablets (18%). (**Table 2**). Not surprisingly, children who did not meet the AAP 2011 television guideline [7] (18%) used all forms of device more frequently than children who met the guidelines.

**Table 2:** Frequency of children’s use of different devices, by whether or not the American Academy of Pediatrics guideline for television viewing was met.

		AAP 2011 guideline*		*P*^†^
	Total (n = 487) n (%)	Meets (n = 86) n (%)	Does not meet (n = 401) n (%)	
*Television*				
Does not use	103 (24%)	53 (62%)	50 (15%)	**<0.001**
Occasional	76 (18%)	27 (31%)	49 (14%)	
Regular	59 (14%)	6 (7%)	53 (15%)	
Daily	191 (44%)	0 (0%)	191 (56%)	
*DVDs or videos*				
Does not use	106 (25%)	51 (66%)	55 (16%)	**<0.001**
Occasional	182 (43%)	25 (32%)	157 (46%)	
Regular	68 (16%)	1 (1%)	67 (19%)	
Daily	65 (15%)	0 (0%)	65 (19%)	
*Computer & console gaming*				
Does not use	116 (27%)	40 (46%)	76 (22%)	**<0.001**
Occasional	191 (45%)	36 (42%)	155 (46%)	
Regular	59 (14%)	10 (12%)	49 (14%)	
Daily	60 (14%)	0 (0%)	60 (18%)	
*Mobile phone*				
Does not use	350 (83%)	71 (93%)	279 (81%)	**0.009**
Occasional	52 (12%)	4 (5%)	48 (14%)	
Regular	12 (3%)	1 (1%)	11 (3%)	
Daily	6 (1%)	0 (0%)	6 (2%)	
*iPad or other tablet*				
Does not use	344 (82%)	71 (93%)	273 (79%)	**0.003**
Occasional	44 (11%)	4 (5%)	40 (12%)	
Regular	14 (3%)	1 (1%)	13 (4%)	
Daily	19 (5%)	0 (0%)	19 (6%)	

* Meets the American Academy of Pediatrics (AAP) guideline [7] if the child watched no television, does not meet the guideline if the child watched any television.

^†^ Mann-Whitney test for rank of ordinal variables.

Unadjusted models found higher maternal pre-pregnancy BMI (1 minute per unit BMI, *P* = 0.013) and maternal screen time (6 minutes per hour of maternal screen time, *P* < 0.001) were positively associated with child television viewing time. Mothers depressed in pregnancy had children who watched an extra 11 minutes of TV per day compared to children of non-depressed mothers (*P* = 0.030). Child’s physical activity (*P* = 0.014) was negatively associated with children’s viewing time (**Table 3**).

**Table 3:** Association between demographic, anthropometric, physical activity, screen time, and depression variables and child television viewing at 24 months of age.

		*Unadjusted model*		
		ß*	(95% CI)	*P*
***Child variables***				
Sex (n = 487)	*Female*	ref		
	*Male*	8.6	(-0.2, 17.3)	0.056
Ethnicity (n = 487)	*New Zealand European*	ref		
	*Māori*	4.3	(-10.3, 18.9)	
	*Other*	-3.8	(-16.5, 9.0)	0.686^†^
BMI z-score (n = 478)		2.8	(-1.7, 7.2)	0.223
Physical activity (100cpm)^‡^ (n = 215)		-7.3	(-13,1, -1.5)	**0.014**
***Maternal variables***				
Age at birth (years) (n = 487)		-0.7	(-1.7, 0.4)	0.205
Ethnicity (n = 487)	*New Zealand European*	ref		
	*Māori*	8.6	(-10.1, 27.2)	
	*Other*	8.6	(-5.4, 22.5)	0.344^†^
Pre-pregnancy BMI (kg/m^2^) (n = 487)		1.0	(0.2, 1.8)	**0.013**
BMI at 24 months (kg/m^2^) (n = 371)		0.7	(-0.1, 1.5)	0.075
Physical activity at 24 months (MET mins^-1^) (n = 372)		0.0	(-0.0, 0.0)	0.299
Screen time (mins/d) (n = 484)		0.1	(0.1, 0.2)	**<0.001**
Maternal depression in pregnancy (n = 486)	*Not depressed*	ref		
	*Possibly depressed*	10.7	(1.01, 20.4)	**0.030**
Parity (n = 487)	*First child*	ref		
	*Subsequent child*	-4.3	(-12.8, 4.2)	0.323
***Partner variables***				
Age at birth (years) (n = 401)		-0.2	(-0.6, 0.3)	0.481
Ethnicity (n = 487)	*New Zealand European*	ref		
	*Māori*	0.0	(-19.2, 19.2)	
	*Other*	8.6	(-0.9, 18.1)	0.202^†^
BMI at baseline (kg/m^2^) (n = 363)		0.7	(-0.3, 1.8)	0.154
BMI at 24 months (kg/m^2^) (n = 119)		1.2	(-0.2, 2.5)	0.089
***Household variables***				
Household deprivation**	*1–3 (Low)*	ref		
(n = 480)	*4–7*	-3.6	(-13.4, 6.3)	
	*8–10 (High)*	0.7	(-11.8, 13.2)	0.700^†^
POI study group	*Control*	ref		
(n = 487)	*FAB*	0.0	(-13.8, 13.8)	
	*Sleep*	-10.0	(-24.3, 4.3)	
	*Combination*	-12.9	(-27.1, 1.4)	0.172^†^

BMI: Body Mass Index; FAB: Food, activity and breastfeeding intervention

* Quantile regression (ß): predicted change to median minutes of children’s television viewing per day for every unit change in independent variable.

^†^ Wald test.

^‡^ From the accelerometry data, refers to 100 counts per minute.

** Salmond C. *et al* [32].

n = number included in the quantile regression analysis

ref = reference group.

There were significant associations between authoritative, authoritarian, and permissive parenting styles and child television viewing in the unadjusted models (**Table 4**). However, only the positive association between the mother having an authoritarian or permissive style, and the partner having an authoritarian style, remained statistically significantly associated with minutes of television watching in adjusted models (**Table 4**). In the current sample, mothers (and partners) with the more authoritarian parenting style had children who watched a median of 17 (and 14) extra minutes of television per day compared to children with less authoritarian parents. More permissive mothers had children who watched a median of an extra 10 minutes of television a day.

**Table 4:** Association between parenting style[41] and child television viewing at 24 months of age.

			Unadjusted model		Adjusted model*	
	n	Median (IQR)	ß (95% CI)^†^	*P*	ß (95% CI)^†^	*P*
Maternal style						
*Authoritative*	429	4.3 (0.7)	-9.3 (-18.5, -0.1)	**0.048**	-7.5 (-16.9, 1.9)	0.119
*Authoritarian*	413	1.5 (0.6)	17.2 (6.3, 28.2)	**0.002**	16.5 (6.1, 27.1)	**0.002**
*Permissive*	434	2.3 (0.8)	14.9 (8.6, 21.1)	**<0.001**	9.9 (2.5, 17.3)	**0.009**
Partner style						
*Authoritative*	277	4.0 (0.7)	-0.0 (-11.3, 11.3)	1.000	-4.3 (-16.2, 7.7)	0.484
*Authoritarian*	273	1.7 (0.6)	17.4 (6.7, 28.1)	**0.001**	14.0 (2.4, 25.7)	**0.018**
*Permissive*	286	2.0 (1.0)	8.6 (1.4, 15.8)	**0.020**	5.6 (-2.4, 13.6)	0.171

* Models were adjusted for items from unadjusted models in Table 3 if *P*<0.25 (maternal analyses: child’s sex, child’s BMI z-score, maternal age at birth, maternal ethnicity, maternal pregnancy BMI, maternal screen time, maternal depression at baseline; partner analyses: child’s sex, child’s BMI z-score, partner ethnicity, paternal BMI at 24 months), with additional adjustment for POI study group, and household deprivation category and maternal parity (variables used for the stratified randomization of the POI participants into groups).

^†^ Quantile regression (ß)—predicted change to median minutes of children’s television viewing per day for every unit change in the parenting style variable.

Although being perceived as being more emotional (by mother), more fussy with food (by mother or partner), or more outgoing (by partner) was associated with watching more television at 24 months of age in the unadjusted models, no infant temperament variables remained statistically significant after adjustment for potential confounders (**Table 5**).

**Table 5:** Association between child temperament [42] and child television viewing at 24 months of age.

			Unadjusted model		Adjusted model*	
	n	Median (IQR)	ß (95% CI)^†^	*P*	ß (95% CI)^†^	*P*
Maternal report						
*Sociability*	469	18 (6)	0.9 (-0.0, 1.9)	0.054	0.6 (-0.4, 1.6)	0.262
*Emotionality*	468	11 (6)	1.6 (0.6, 2.7)	**0.002**	1.0 (-0.1, 2.0)	0.085
*Activity*	469	21 (5)	0.3 (-1.0, 1.6)	0.616	0.3 (-1.0, 1.6)	0.676
*Attention span—persistence*	469	16 (4)	0.3 (-0.9, 1.5)	0.606	0.1 (-1.4, 1.1)	0.823
*Reaction to food*	468	11 (7)	1.4 (0.4, 2.3)	**0.004**	0.9 (-0.1, 1.8)	0.091
*Soothability*	469	17 (5)	-1.0 (-2.3, 0.4)	0.157	-0.3 (-1.7, 1.1)	0.672
Paternal report						
*Sociability*	285	19 (5)	1.7 (0.4, 3.1)	**0.014**	1.0 (-0.6, 2.5)	0.217
*Emotionality*	285	11 (5)	1.0 (-0.6, 2.5)	0.217	0.4 (-1.1, 1.9)	0.605
*Activity*	285	21 (4)	-1.2 (-3.1, 0.6)	0.191	-0.2 (-2.2, 1.8)	0.844
*Attention span—persistence*	284	16 (4)	1.0 (-0.9, 2.9)	0.299	0.8 (-1.2, 2.8)	0.418
*Reaction to food*	287	12 (6)	1.3 (0.1, 2.4)	**0.036**	1.0 (-0.1, 2.2)	0.079
*Soothability*	285	17 (4)	-1.3 (-3.1, 0.6)	0.171	-1.0 (-3.0, 1.0)	0.310

* Models were adjusted for items from unadjusted models in Table 3 if *P*<0.25 (maternal analyses: child’s sex, child’s BMI z-score, maternal age at birth, maternal ethnicity, maternal pregnancy BMI, maternal screen time, maternal depression at baseline; paternal analyses: child’s sex, child’s BMI z-score, paternal ethnicity, paternal BMI at 24 months), with additional adjustment for POI study groups, and household deprivation category [32] and maternal parity (variables used for the stratified randomization of the POI participants into groups).

^**†**^ Quantile regression (ß)—predicted change to median minutes of children’s television viewing per day for every unit change in the infant temperament variable.

The mother reporting that their family was “active or sporty” (negative relationship), or “musical” (positive relationship) was associated with television watching in unadjusted models, but neither of these relationships remained significant after adjusting for potential confounders (**S1 Table**). By contrast, the partner reporting that their family was “active or sporty” was associated with significantly fewer hours of television viewing by toddlers in both models (adjusted model: 29 minutes less, *P* = 0.002), whereas the partner reporting that their family was “media savvy” was associated with significantly more television viewing per day in both models (adjusted model: 20 minutes more, *P* = 0.049) (**S2 Table**). The partner reporting that their family was “outdoor” was associated with 11 fewer minutes of television watching per day (*P* = 0.011) in the adjusted model.

## Discussion

Our study investigated associations between parenting style, infant temperament, and family type, and television viewing in a large community sample of two-year old children. Parents who scored higher on the authoritarian or permissive parenting scales had children who viewed approximately quarter of an hour more television each day, even after adjustment for confounding variables. Although several components of infant temperament were initially associated with television viewing, these were no longer significant after adjustment. Partner description of family type was also associated with either less (“active or sporty” or “outdoor” families) or more (“media savvy” families) television viewing at this age.

Our findings regarding parenting style are in agreement with the limited existing research in older children. More television viewing has been observed in children of more permissive mothers [45], and in those who valued complete compliance, that is, having children who did as they were told [15]. Parents who are more permissive are likely to give their child more freedom to do what they like, so it is of little surprise that their children watch more television. Authoritarian parents are more likely to be angry towards their child, to be openly critical, and to use criticism to improve behavior [41], so it is possible that a child avoids these parental responses when they are watching television quietly, or perhaps authoritarian parents use television as a coping strategy. Another possible explanation is low parental involvement as authoritarian and permissive parents may be similar in their relative detachment, and in the ineffectiveness of their communication [23]. Low parental involvement has been found to be associated with high screen use in pre-schoolers [46], and interventions aimed at increasing parent-child interactions and parental stimulation in the home have significantly reduced screen time compared to controls [47, 48].

In our study, unadjusted analyses suggested an association between television watching and mothers perceiving that their toddler got upset more easily, their other parent thinking that the toddler was more sociable, and either parent believing the toddler was fussy about food. However, these differences were very small (less than 2 minutes) and were no longer significant once potential confounders had been controlled for in the multivariate model. Other research has indicated that young children who are considered to be fussy by their parents may watch more television, perhaps as a coping strategy for parents [17]. Infants from low-income homes whose mothers believed they were fussy were 23% more likely to be exposed to at least one hour of television per day [17]. Similarly, infants classified as having poor self-regulation (defined as being unpredictably fussy, or having problems with sleep, feeding, or regulating mood and behavior) at 9 months of age watched 14 more minutes of television at two years of age [24]. The observation that these relationships were stronger in those with lower socioeconomic status [24] may explain the lack of effect in our study, given our sample was well educated and had relatively high socioeconomic status.

Parental description of family type was strongly associated with television viewing in the current study: children from “media-savvy” families watched about 20 more minutes per day, with children from “active or sporty” families watching half an hour less, and those from “outdoor” families watching 14 minutes less. Our findings are in agreement with a number of studies that have reported an association between access to media, and screen viewing time in young children [49]. However, relationships between physical activity and television viewing appear more inconsistent [50]. It is interesting in this context that, in the current study, toddler physical activity, and the two most active family types, were all associated with less television viewing.

Children in the current study had considerably lower television viewing times than are typically reported in the literature (21 minutes compared to 1.3–3.6 hours per day) [4, 10, 15, 16]. This may be because very young children in New Zealand watch less television. Certainly one study has suggested that New Zealand toddlers may be watching as little as 1–3 hours per week [11], although other New Zealand data suggest children this age could be watching 90 minutes of television a day [12]. Thus is more likely to be a reflection of differences in the way viewing time is assessed. Alternatives range from assessing television watching based on discrete hourly response options [15], to asking parents to report how long their child watches each of several different types of content [10], both of which approaches may over-estimate total viewing time. In addition, existing studies in preschoolers include a wide age range (0–5 years), with lower levels of television viewing (46 minutes per day) having been reported in children aged 1–2 years [21]. Whether background television is included in the measurement is also important, given that children under the age of 24 months have been reported to be exposed to very high levels (5.5 hours per day in one study [51]). Our viewing times were specific to the child’s viewing, but mothers reported that the television was on in the background for 210 minutes per day.

The strengths of our study include the examination of parenting style, infant temperament and family type in relation to toddler television viewing in a large cohort of families, with comprehensive assessment of a variety of measures which allowed many factors to be investigated as potential confounders. The main limitation of our study was its reliance on maternal report of usual television viewing in toddlers. Although we cannot be sure that the values reported always refer to times when the child was actively watching television, rather than background television, this seems unlikely given the low median viewing times in our sample. Previous research has found negative outcomes for both forms of exposure to television [52], and parental reports of children’s television viewing have been shown to be highly correlated with media time when measured by video recording in the home [53, 54]. We were also only able to include television viewing as our outcome variable which may under-estimate the total screen time toddlers were exposed to. However, we were able to provide insight into the frequency of other media use, which was generally less than that of television. We acknowledge that our family type questionnaire is not validated as such, but were interested in examining how both parents viewed their family type to provide a more holistic view of family life. Finally, we were unable to control for other potential confounders, such as child’s physical activity, paternal screen time, day care attendance, and number of siblings. However, parity (a proxy for number of siblings) was examined, but was not included in the adjusted models as it did not reach the required significance level. Exclusion of those participants who refused physical activity data from the current study likely biases our results towards the positive and inflates the magnitude of our associations. This is because there is likely residual confounding between physical activity and TV watching, as those excluded may have watched more television, as indicated by our significant result between child’s physical activity and daily TV viewing. However, all studies of this nature are likely to have some degree of residual confounding.

In conclusion, our study shows parenting style and family type are associated with television watching in young children. Future longitudinal research needs to examine associations between the evolution of parenting style as the child ages and screen time in children to determine whether these associations persist, ideally in a more economically diverse sample. Parenting style is not necessarily modifiable, but future interventions are likely to need to understand the impact of parenting style in order to assist families with effective strategies for encouraging activity and reducing screen time. Further research is also required to determine the extent to which family type is modifiable, and whether any specific characteristics of family type are both related to television viewing, and transferable.

## Supporting information

S1 Table Associations between family type as indicated by the mother and child television viewing at 24 months of age. * Models were adjusted for items from unadjusted models in Table 3 if *P*<0.25 (child’s sex, child’s BMI z-score, maternal age at birth, maternal ethnicity, maternal pregnancy BMI, maternal screen time), with additional adjustment for POI study groups and NZ Deprivation category [32] and maternal parity (variables used for the stratified randomization of the POI participants into groups).
^†^ Quantile regression (ß): predicted change to median minutes of children’s television viewing per day between reference and non-reference levels of categorical of family type.
^‡^ Wald test.
** Reference group.
(DOCX)

S2 Table Associations between family type as indicated by the partner and child television viewing at 24 months of age. * Models were adjusted for items from unadjusted models in Table 3 if *P*<0.25 (child’s sex, child’s BMI z-score, maternal age at birth, maternal ethnicity, maternal pregnancy BMI, maternal screen time), with additional adjustment for POI study group and household deprivation category [32] and maternal parity (variables used for the stratified randomization of the POI participants into groups).
^†^ Quantile regression (ß): predicted change to median minutes of children’s television viewing per day between reference and non-reference levels of categorical of family type.
^‡^ Wald test.
** Reference group.
(DOCX)
