# Supplementary material for: Parenting style and family type, but not child temperament, are associated with television viewing time in children at two years of age
Source: PLoS One. 2017 Dec 20;12(12):e0188558. doi: 10.1371/journal.pone.0188558 (PMC5737952; doi:10.1371/journal.pone.0188558)
Supplement: S1 Table — * Models were adjusted for items from unadjusted models in Table 3 if P<0.25 (child’s sex, child’s BMI z-score, maternal age at birth, maternal ethnicity, maternal pregnancy BMI, maternal screen time), with additional adjustment for POI study groups and NZ Deprivation category [32] and maternal parity (variables used for the stratified randomization of the POI participants into groups). † Quantile regression (ß): predicted change to median minutes of children’s television viewing per day between reference and non-reference levels of categorical of family type. ‡ Wald test. ** Reference group. (DOCX) [file pone.0188558.s001.docx]

**S1 Table.** Associations between family type as indicated by the mother and child television viewing at 24 months of age

|  |  | **Unadjusted model** | | **Adjusted model^*^** | |
| --- | --- | --- | --- | --- | --- |
|  | **n (%)** | **ß (95% CI)^†^** | ***P*^‡^** | **ß (95% CI)^†^** | ***P*^‡^** |
| Active or sporty |  |  |  |  |  |
| *Not really like us*^**^ | 89 (19) | - |  | - |  |
| *A little like us* | 235 (50) | 0.0 (-12.9, 12.9) |  | -3.2 (-16.1, 9.7) |  |
| *Definitely like us* | 146 (31) | -12.9 (-26.7, 1.0) | **0.048** | -9.0 (-23.1, 5.2) | 0.410 |
| Media savvy |  |  |  |  |  |
| *Not really like us*^**^ | 131 (28) | - |  | - |  |
| *A little like us* | 222 (47) | 4.3 (-6.3, 14.9) |  | 6.8 (-4.2, 17.7) |  |
| *Definitely like us* | 118 (25) | 12.9 (0.5, 25.2) | 0.114 | 13.1 (0.4, 25.7) | 0.127 |
| Bookish |  |  |  |  |  |
| *Not really like us*^**^ | 46 (10) | - |  | - |  |
| *A little like us* | 226 (48) | -10.7 (-26.7, 4.3) |  | -6.6 (-21.5, 8.3) |  |
| *Definitely like us* | 199 (42) | -10.7 (-25.8, 4.4) | 0.343 | -4.2 (-19.4, 11.0) | 0.653 |
| Outdoor people |  |  |  |  |  |
| *Not really like us*^**^ | 51 (11) | - |  | - |  |
| *A little like us* | 236 (50) | 0.0 (-18.8, 18.8) |  | -5.7 (-21.3, 9.9) |  |
| *Definitely like us* | 184 (39) | -12.9 (-32.1, 6.4) | 0.086 | -14.2 (30.2, 1.8) | 0.118 |
| Musical |  |  |  |  |  |
| *Not really like us*^**^ | 150 (32) | - |  | - |  |
| *A little like us* | 218 (46) | 4.3 (-3.7, 12.3) |  | 5.2 (-5.7, 16.0) |  |
| *Definitely like us* | 103 (22) | 22.9 (13.2, 32.5) | **<0.001** | 15.5 (2.2, 28.9) | 0.073 |
| Religious/spiritual |  |  |  |  |  |
| *Not really like us*^**^ | 343 (73) | - |  | - |  |
| *A little like us* | 83 (18) | 8.6 (-3.8, 20.9) |  | 9.6 (-2.1, 21.4) |  |
| *Definitely like us* | 45 (10) | -7.1 (-23.0, 8.7) | 0.216 | -3.1 (-18.9, 12.7) | 0.217 |
| Creative or arty |  |  |  |  |  |
| *Not really like us*^**^ | 126 (27) | - |  | - |  |
| *A little like us* | 241 (51) | -1.4 (-10.1, 7.3) |  | -3.2 (-14.1 7.6) |  |
| *Definitely like us* | 104 (22) | 8.6 (-1.9, 19.0) | 0.094 | 6.4 (-6.7, 19.4) | 0.265 |

^*^ Models were adjusted for items from unadjusted models in Table 3 if *P*<0.25 (child’s sex, child’s BMI z-score, maternal age at birth, maternal ethnicity, maternal pregnancy BMI, maternal screen time), with additional adjustment for POI study groups and NZ Deprivation category(25) and maternal parity (variables used for the stratified randomization of the POI participants into groups).

^†^ Quantile regression (ß): predicted change to median minutes of children’s television viewing per day between reference and non-reference levels of categorical of family type.

^‡^ Wald test.

^**^ Reference group.
